# Medical Clinic: Diseases of the Abdomen

**Published:** 1843-07-22

**Authors:** 


### BIBLIOGRAPHICAL NOTICES.

1. *Medical Clinic: Diseases of the Abdomen.* By G. Andral, Professor of the Faculty of Medicine of Paris, &c. &c. *Condensed and Translated, with Observations.* By D. Spillan, M. D., &c. &c. Philadelphia: Ed. Barrington & George D. Haswell. 1843. 8vo. pp. 420.

